# LncRNA FAM83H-AS1 promotes the malignant progression of pancreatic ductal adenocarcinoma by stabilizing FAM83H mRNA to protect β-catenin from degradation

**DOI:** 10.1186/s13046-022-02491-2

**Published:** 2022-09-29

**Authors:** Min Zhou, Shutao Pan, Tingting Qin, Chunle Zhao, Taoyuan Yin, Yang Gao, Yuhui Liu, Zhenxiong Zhang, Yongkang Shi, Yu Bai, Jun Gong, Xingjun Guo, Min Wang, Renyi Qin

**Affiliations:** Department of Biliary-Pancreatic Surgery, Affiliated Tongji Hospital, Tongji Medical College, Huazhong University of Science and Technology, 1095 Jiefang Ave, Wuhan, 430030 Hubei China

**Keywords:** Pancreatic ductal adenocarcinoma, FAM83H-AS1, FAM83H, mRNA stability, β-catenin, Metastasis

## Abstract

**Background:**

Pancreatic ductal adenocarcinoma is prone to metastasis, resulting in short survival and low quality of life. LncRNAs are pivotal orchestrators that participate in various tumor progress. The underlying role and mechanism of lncRNA FAM83H-AS1 is still unknown in PDAC progression.

**Methods:**

To address this issue, firstly, we profiled and analyzed the aberrant lncRNA expression in TCGA database and identified FAM83H-AS1 as the most effective one in promoting the migration of pancreatic cancer cells. Then, the expression levels of FAM83H-AS1 in patient’s serum, tumor tissues and PDAC cells were detected using RT-qPCR, and FAM83H-AS1 distribution in PDAC cells was determined by performing FISH and RT-qPCR. Next, a series of in vivo and in vitro functional assays were conducted to elucidate the role of FAM83H-AS1 in cell growth and metastasis in PDAC. The regulatory relationship between FAM83H-AS1 and FAM83H (the homologous gene of FAM83H-AS1) was verified by performing protein and RNA degradation assays respectively. Co-IP assays were performed to explore the potential regulatory mechanism of FAM83H to β-catenin. Rescue assays were performed to validate the regulation of the FAM83H-AS1/FAM83H/β-catenin axis in PDAC progression.

**Results:**

FAM83H-AS1 was highly expressed in the tumor tissues and serum of patients with PDAC, and was correlated with shorter survival. FAM83H-AS1 significantly promoted the proliferation, invasion and metastasis of PDAC cells, by protecting FAM83H mRNA from degradation. Importantly, FAM83H protein manifested the similar malignant functions as that of FAM83H-AS1 in PDAC cells, and could bind to β-catenin. Specifically, FAM83H could decrease the ubiquitylation of β-catenin, and accordingly activated the effector genes of Wnt/β-catenin signaling.

**Conclusions:**

Collectively, FAM83H-AS1 could promote FAM83H expression by stabilizing its mRNA, allowing FAM83H to decrease the ubiquitylation of β-catenin, thus resulted in an amplified FAM83H-AS1/FAM83H/β-catenin signal axis to promote PDAC progression. FAM83H-AS1 might be a novel prognostic and therapeutic target for combating PDAC.

**Supplementary Information:**

The online version contains supplementary material available at 10.1186/s13046-022-02491-2.

## Background

Pancreatic cancer is one of the top four leading causes of cancer-related death and is projected to surpass breast, prostate, and colorectal cancers to rise to third or second place [1]. Because this disease is commonly diagnosed at an advanced stage, the 5-year survival rate of patients with pancreatic cancer is only approximately 10% [2–4]. Among the various types of pancreatic cancer, pancreatic ductal adenocarcinoma (PDAC) comprises the vast majority of cases [5]. The modest advances achieved in PDAC therapy in recent years are due to comprehensive treatment strategies, especially those based on biomarkers and personalized therapy [6]. Even with a better understanding of PDAC pathophysiology and advances in treatment strategies, the slight improvement in outcomes has not substantially increased the survival time [7]. Researchers hope that molecular targeted therapy for PDAC will lead to greater progress in the near future.

Long noncoding RNAs (lncRNAs), once considered genomic noise, have been shown to account for a considerable portion of the functional genome [8]. LncRNAs have been reported to participate in numerous vital biological processes, including imprinting genomic loci, shaping chromosome conformation, and allosterically regulating enzymatic activity [9]. Dysfunction of lncRNAs is commonly implicated in numerous human diseases, especially cancers [10]. LncRNAs have been learned to be as key regulators of cancer pathways and also as biomarkers of metastatic diseases [11–13]. lncRNA MALAT1 is widely studied and important for cancer-associated pathways regulation, which includes MAPK/ERK, PI3K/AKT, Wnt/β-catenin, Hippo, VEGF, YAP, etc. [14]. LncRNA ZEB1-AS1 epigenetically regulates the homologous sense gene ZEB1 to promote the progression of prostate cancer [15]. As they intersect with exons in the protein-coding locus on the other strand, antisense lncRNAs have an inherent capacity to regulate their corresponding sense genes at the transcriptional and/or post-transcriptional levels [15, 16].

Since the first member of the Wnt family was discovered four decades ago, interest in the Wnt signaling pathway has been constantly increasing [17]. As a fundamental growth control pathway, Wnt signaling has been analyzed in contexts ranging from early animal evolution to cancer and development [17]. The growth-related effectors of Wnt signaling play vital roles in intercellular communication, including the regulation of cell fate, proliferation and stem cell maintenance [18]. In colorectal cancer, canonical Wnt/β-catenin signaling is the main form of Wnt signaling, which is induced by 5% *CTNNB1* and 73% *APC* mutations [19, 20]. The dual effects of canonical and noncanonical Wnt signaling are widely learned in breast cancers [21, 22]. Accumulating studies have manifested that β-catenin regulates the maintenance and progression of PDAC [23, 24]. The main effector of Wnt signaling, β-catenin, regulates the transcription of Wnt effector genes by translocating from the cytoplasm to the nucleus and binding to TCF/Lef transcription factors [25, 26]. In epithelia, β-catenin also indispensably binds to the cytoplasmic tail of various cadherins, such as E-cadherin, in adherens junctions. β-catenin is highly associated with the epithelial-mesenchymal transition (EMT) in human cancers [27–29].

In this study, we screened for lncRNAs associated with the metastasis of PDAC in The Cancer Genome Atlas (TCGA) database and finally focused on FAM83H-AS1, the most prominent lncRNA, which was verified by functional assays and survival analysis. Furthermore, a series of functional assays were conducted in vitro and in vivo to assess its biological functions in PDAC. Mechanistically, we revealed that FAM83H-AS1 protected FAM83H mRNA from degradation and that FAM83H bound to β-catenin to mitigate its ubiquitylation and subsequent degradation. As a result, the accumulation of β-catenin induced the expression of the effector genes of Wnt/β-catenin signaling. Taken together, the results of this study revealed the oncogenic function of the FAM83H-AS1/FAM83H/β-catenin axis in the progression of PDAC. Furthermore, the FAM83H-AS1/FAM83H/β-catenin axis may be a promising biomarker and potential therapeutic target for PDAC.

## Materials and methods

### Clinical sample collection and ethics statement

PDAC tissue samples (*n* = 89) and paired adjacent noncancerous tissue samples (*n* = 89) were harvested from patients with PDAC who had undergone surgical resection at Tongji Hospital of Huazhong University of Science and Technology (Wuhan, China) between January 2016 and August 2018. The basic clinical characteristics of patients whose tissues were collected for analysis were listed in Supplementary file 4: Table S4. All samples were immediately snap frozen in liquid nitrogen and stored at -80 °C after being dissected, and were validated by two independent experienced pathologists. Human serum was collected from patients with PDAC (*n* = 57), benign nonpancreatic disease (*n* = 39) and benign pancreatic disease (*n* = 21) before the operation using a 21-gauge needle push button blood collection and finally transferred to 8 mL serum separator clot activator vacuum tubes. The basic clinical characteristics of patients whose serum samples were collected were listed in Supplementary file 3: Table S3. The tubes were inverted 10 times before the samples were allowed to clot for 30 min. The tubes were centrifuged for 15 min at 1,200 g in a swinging bucket rotor. Serum was removed from the tube and aliquoted into 500 μL volumes in 1.5 mL microcentrifuge tubes. Serum was used immediately or frozen at -80 °C for future use. Informed consent forms were signed by all patients who provided tissue and serum samples, and the study was approved by the Human Ethics Committee of Tongji Hospital of Huazhong University of Science and Technology (Wuhan, China) and conducted strictly according to the principles of the Declaration of Helsinki.

### Cell culture

Seven human PDAC cell lines (MIA PaCa-2, PANC-1, SW 1990, Capan-2, BxPC-3, CFPAC-1 and Panc 03.27), an immortalized human pancreatic duct epithelial (HPDE) cell line and HEK293T cells were purchased from the American Type Culture Collection (ATCC, Manassas, VA, USA). All cell lines were authenticated by short tandem repeat (STR) profiling and confirmed to be mycoplasma-free by testing. HEK293T, PANC-1, MIA PaCa-2, SW 1990 and CFPAC-1 cells were all cultured in DMEM (Gibco, NY, USA), while Capan-2, Panc 03.27, BxPC-3 and HPDE cells were cultured in RPMI-1640 medium (Gibco, NY, USA) with 10% fetal bovine serum (FBS, Gibco, NY, USA) in a 5% CO_2_ atmosphere at 37 °C.

### Plasmid construction and cell transfection

The full-length human FAM83H and CTNNB1 (β-catenin) cDNAs were synthesized and cloned into the expression vector pHAGE-puro plasmid (Invitrogen, China) with a FLAG-tag and HA-tag, respectively. The plasmid expressing Myc-Ub expression was maintained in the Biliary-Pancreatic Surgery laboratory. The lentivirus overexpressing FAM83H-AS1 and the negative control were synthesized by and purchased from GeneChem (Shanghai, China). Small hairpin RNAs (shRNAs) targeting human FAM83H-AS1 and FAM83H were synthesized and cloned into the pLKO.1-puro plasmid (Invitrogen, China). All plasmids and siRNAs were transfected into human PDAC cells using Lipofectamine 3000 (Invitrogen, USA) following the manufacturer’s instructions. All shRNA sequences and siRNA sequences are listed in Supplementary file 1: Table S1.

### Immunofluorescence (IF) staining

Cells were evenly dispensed on a single glass coverslip placed in 6-well plates. The cells were fixed with 4% formaldehyde for 35 min and permeabilized with 0.1% Triton X-100 (Sigma-Aldrich, Germany) for 8 min. After the fixed cells were blocked with 5% BSA for 35 min at room temperature, they were incubated with the primary antibody overnight at 4 °C on an oscillating table. The primary rabbit polyclonal antibody FAM83H (Novus, NBP1-93,737) was diluted with 5% BSA at 1:50. After incubation with the Cy3-conjugated secondary antibody for 1 h at room temperature, the samples were counterstained with DAPI (Boster Biological Technology, Wuhan, China) for 10 min. Cell photographs were then captured with a fluorescence microscope (Olympus, Japan).

### RNA fluorescence in situ hybridization (FISH)

Briefly, the cells were incubated with horseradish peroxidase (HRP)-conjugated anti-DIG to detect the LNA probe signals, which were then amplified using tetramethylrhodamine (TRITC)-conjugated tyramide. The signals were determined using a tyramide signal amplification (PerkinElmer, USA) system to determine FAM83H-AS1 expression in cells, and the images were captured using a fluorescence microscope (Olympus, Japan).

### Cell Counting Kit-8 (CCK-8) assay

Cell viability was assessed with the CCK-8 assay (Yeasen, Shanghai, China) following the manufacturer’s instructions. Briefly, cells were seeded in a 96-well plate at a density of 1 × 10^4^–1 × 10^5^ cells/well in 100 μL of culture medium with 10% FBS (incubated in a humidified atmosphere at 37 °C with 5% CO_2_). Ten microliters of CCK-8 solution were added to each well and incubated for 1.5 h/2.0 h in the incubator. Differences in shades of orange represent the various numbers of living cells in each well. The samples were mixed gently on an orbital shaker for 1 min to ensure the homogeneous distribution of color, and then, the absorbance was measured at 450 nm with a microplate reader (Thermo Scientific, USA).

### EdU staining assay

Cells were seeded in 6-well plates and incubated until the logarithmic growth phase. EdU (5-ethynyl-2’-deoxyuridine) solution (Beyotime Biotechnology, Shanghai, China) was added to the medium of each plate and incubated for 2 h. After the medium was removed, the cells were fixed with 4% paraformaldehyde for 15 min and imaged by fluorescence microscopy. Cell viability was measured by the EdU staining positive rates compared to that of the control group in accordance with the manufacturer’s instructions.

### Scratch wound-healing assay

Cells were evenly dispensed in 6-well plates and cultured to 90% confluence. Pipette tips (200 μL) were used to create scratch wounds. The detached cells were then removed by PBS washes, and the remaining cell sheets were cultured in basal medium (1% FBS) for another 48 h. The wound was photographed with a phase contrast microscope (Nikon, Japan), and the closure rates of the wounded areas were measured with ImageJ software.

### Colony formation assay

Cell suspensions (4 mL, 1000 or 2000 cells/well) were evenly dispensed in 6-cm dishes and incubated for half a month in an incubator (humidified atmosphere, 37 °C, and 5% CO_2_). The cells were then fixed with 4% paraformaldehyde for 35 min and stained with 0.1% crystal violet. Cell viability was assessed by counting the colonies (diameters greater than 100 μm) and determining the significance by performing statistical tests.

### Proliferation assays in vivo

Female BALB/c nude mice aged 4–5 weeks were purchased from Charles River (Beijing, China). For tumorigenicity assays, the indicated stably transfected PANC-1 cells (1 × 10^6^ cells/0.2 mL of PBS) were subcutaneously injected into the right flank of each mouse. Tumor sizes and mouse weights were measured once per week, and mice were sacrificed to analyze the tumor burden after 4 weeks. The tumor volume was calculated with the following formula: V = (length × width^2^)/2. The tumors were then embedded in paraffin and cut into sections with a thickness of 4 μm. The sections were stained with hematoxylin and incubated with primary Ki-67 antibodies (Abcam, catalog no. ab15580) and proliferating cell nuclear antigen (PCNA, Servicebio, catalog no. GB11010) using the Polymer HRP (Mouse/Rabbit) IHC Kit (Maxim, Fujian, China). Images of representative fields were obtained with a phase contrast microscope (Nikon, Japan).

### Metastasis assays in vivo

For metastasis assays, the indicated and stably transfected SW 1990 cells (2 × 10^6^ cells/0.2 mL of PBS) were injected into nude mice via the tail vein. During the period of tumor metastasis, bioluminescence imaging was performed to observe the metastatic conditions. For imaging, 100 mg/kg luciferin was intraperitoneally injected into mice 10 min prior to imaging with an IVIS Spectrum Imaging System (PerkinElmer). Imaging settings remained the same throughout the study, and luminescence intensity was measured using Living Image Software (PerkinElmer). Mouse weights were measured each week for 10 weeks. During the observation period, the mice constantly died due to cachexia. At the endpoint time of 14 weeks, all the mice were sacrificed, and the lungs were surgically excised. The lung tissues were embedded in paraffin for hematoxylin and eosin (H&E) staining and statistical analysis of the number of tumor nodules. All animal experiments mentioned above were conducted with the approval of the Institutional Animal Care Committee of Tongji Hospital, Tongji Medical College, Huazhong University of Science and Technology.

### Western blot and immunoprecipitation assays

Cells were harvested and lysed with enhanced RIPA buffer (Boster Biological Technology, Wuhan, China) containing protease and phosphatase inhibitors (F. Hoffmann-La Roche Ltd, Switzerland). Lysates were centrifuged at 12,000 g at 4 °C for 15 min. For immunoprecipitation, cells were harvested from 10 cm dishes and lysed with 500 μL of IP lysis buffer on ice for 30 min. After ultrasonication and centrifugation (12,000 g, 15 min, 4 °C), the supernatant was separated into 50 μL (for input) and 450 μL (for IP) aliquots. Ten microliters of anti-FLAG/anti-HA magnetic beads were added to 450 μL of lysate and incubated overnight at 4 °C. Thereafter, the precipitates were washed 3–4 times with IP buffer, and 40–60 μL of loading dye (diluted to 1 × IP buffer) was added to the immune complexes and then boiled. Total proteins were separated on SDS‒PAGE gels and transferred onto PVDF membranes (Millipore, MA, USA). Membranes were blocked with 5% skim milk and incubated with primary antibodies at the recommended dilution in TBST with 5% skim milk overnight at 4 °C on an oscillating table. The next day, the membranes were incubated with fluorescently labeled secondary antibodies at the recommended dilution and imaged using a ChemiDoc XRS + System (Bio-Rad Laboratories, USA). Primary antibodies specific for the following proteins were used: β-actin (ABclonal, catalog no. AC026), β-catenin (Proteintech Group, catalog no. 51067–2-AP), FAM83H (Abcam, catalog no. 121828), phospho-Akt (Ser473) (CST, catalog no. 4060 T), AKT1 (ABclonal, catalog no. A16343), E-cadherin (CST, catalog no. 3195P), N-cadherin (CST, catalog no. 13116S), Vimentin (CST, catalog no. 5741P), Notch1 (ABclonal, catalog no. A19019), CCDC88A (ABclonal, catalog no. A16132), and HuR/ELAVL1 (ABclonal, catalog no. A19622). The following fluorescently tagged secondary antibodies were used: HRP-conjugated AffiniPure goat anti-rabbit IgG (Boster Biological Technology, catalog no. BA1054) and HRP-conjugated AffiniPure goat anti-mouse IgG (Boster Biological Technology, catalog no. BA1050).

### In vivo ubiquitylation assay

HEK293T cells were cotransfected with HA-β-catenin, Myc-Ub and sh-FAM83H#1 or sh-FAM83H#2, along with sh-Control, or cotransfected with HA-β-catenin, Myc-Ub and sh-FAM83H-AS1 or FLAG-FAM83H, along with sh-Control. Six hours before harvesting, the cells were treated with MG132 (5 μM). Ubiquitylation assays were conducted using the same immunoprecipitation method described above.

### RNA isolation and reverse transcription quantitative PCR (RT-qPCR)

Total RNA was isolated from PDAC tissues and adjacent tissues or PDAC cells using TRIzol (TaKaRa, Dalian, China) following the manufacturer’s instructions. The isolation of serum RNA also followed the same protocol mentioned above. Complementary DNA (cDNA) was reverse-transcribed from 1 μg of RNA using HiScript III RT SuperMix (Vazyme, Nanjing, China). RT-qPCR was performed using ChamQ SYBR qPCR Master Mix (Vazyme, Nanjing, China) with a CFX Connect PCR system (Bio-Rad Laboratories, USA). GAPDH or β-actin was used as a reference gene. All primers used for RT-qPCR are listed in Supplementary file 2: Table S2.

### Statistical analysis

All statistical analyses were conducted with GraphPad Prism v8.0 software (GraphPad, Inc., La Jolla, CA, USA) and SPSS 22.0 software (SPSS, Inc., IL, USA). All data are presented as the mean ± standard deviation (SD) of at least three independent replicates. Differences between two groups were evaluated by Student’s* t* test, and differences among multiple groups were evaluated by one-way ANOVA. Estimation of survival differences was performed using the Kaplan–Meier method and the log-rank test. The Pearson correlation coefficient was used to evaluate correlations between expression and survival. All *P* values were two-sided. *P* < 0.05 was considered statistically significant.

## Results

### FAM83H-AS1 is the top potential oncogene in a metastasis screening model of PDAC

**Fig. 1:**
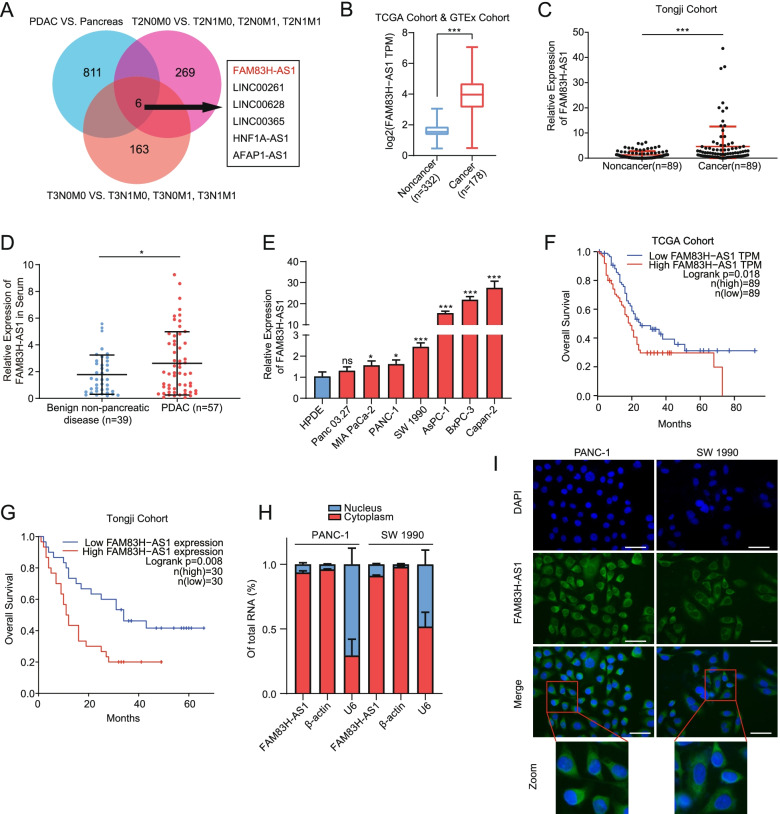
FAM83H-AS1 is upregulated in PDAC tissues and positively correlated with poorer prognosis. **A** Venn diagram of abnormally expressed lncRNAs in three comparison models (PDAC vs. pancreas, T2N0M0 vs. T2N1M0/T2N0M1/T2N1M1 and T3N0M0 vs. T3N1M0/T3N0M1/T3N1M1) based on RNA-seq data from the TCGA database. **B**, **C** FAM83H-AS1 expression in the combined TCGA and GTEx cohort (**B**) and the cohort from Tongji Hospital (**C**). **D** Serum FAM83H-AS1 expression was tested in patients with PDAC and benign nonpancreatic disease from Tongji Hospital. **E** RT–qPCR results showing the expression of FAM83H-AS1 in HPDE and PDAC cell lines (Panc 03.27, MIA PaCa-2, PANC-1, SW 1990, AsPC-1, BxPC-3 and Capan-2). **F**-**G** A survival analysis was conducted to illustrate the relationship between the level of FAM83H-AS1 and the overall survival of patients in the TCGA cohort (**F**) and the cohort from Tongji Hospital (**G**). **H** Nuclear/cytosol fractionation followed by RT-qPCR analysis of the level of FAM83H-AS1 in the nucleus and cytoplasm. **I** Combination of FISH and IF assays showing the subcellular distribution of FAM83H-AS1; scale bar: 50 μm. *P < 0.05 and ***P < 0.001

A metastasis screening model with 3 comparison levels was established to identify aberrantly expressed lncRNAs associated with lymphatic and distant metastasis in PDAC. A total of 817 differentially expressed lncRNAs were identified by comparing the RNA-seq profiles of 4 adjacent tissues and 178 PDAC tissues from the TCGA cohort (Figure S1). In a comparison of the lncRNA expression profiles from the T2N0M0 subgroup with those from the T2N1M0/T2N0M1/T2N1M1 subgroups in the same cohort, 275 aberrantly expressed lncRNAs were identified (Figure S2). The same strategy was applied to compare the T3N0M0 subgroup with the T3N1M0/T3N0M1/T3N1M1 subgroups, and another 169 aberrantly expressed lncRNAs were identified (Figure S3). The Venn diagram revealed that the expression levels of 6 lncRNAs (FAM83H-AS1, LINC00365, LINC00628, LINC00261, AFAP1-AS1, and HNF1A-AS1) were altered in all three models (Fig. 1A). Kaplan–Meier survival analysis indicated that elevated FAM83H-AS1 levels and decreased LINC00261 levels were associated with decreased overall survival (OS) in PDAC patients, while the remaining 4 lncRNAs had no effect on OS (Figure S4). Transwell assays (Figure S5A) and scratch wound-healing assays (Figure S5B) were performed in two PDAC cell lines (PANC-1 and SW 1990) with RNA interference-mediated knockdown of these 6 lncRNAs separately to evaluate the capabilities of these 6 lncRNAs to promote PDAC cell migration. Only FAM83H-AS1 potently promoted PDAC cell migration. Furthermore, we analyzed the effect of these 6 lncRNAs on filamentous actin (F-actin) reorganization in SW 1990 cells by performing phalloidin staining and IF staining. FAM83H-AS1 deficiency resulted in the most prominent depolymerization of F-actin compared with that of the negative control (NC) group (Figure S6). Taken together, these results showed that FAM83H-AS1, a potential oncogene, was involved in the malignant biological behaviors of PDAC.

### FAM83H-AS1 is mainly localized in the cytoplasm, and its upregulation in PDAC predicts a poor prognosis

First, we detected the expression of FAM83H-AS1 in pancreatic nontumor and pancreatic tumor tissues from a combined cohort [TCGA and Genotype-Tissue Expression (GTEx)] (Fig. 1B) and a cohort of patients who underwent surgical resection at Tongji Hospital (Tongji cohort) (Fig. 1C). The results consistently showed significantly higher FAM83H-AS1 expression in the PDAC tissues than in the nontumor tissues. Given the important role of lncRNAs in the pathological process of cancers, their circulating abundance, which is easily detected, would be a prerequisite for a potential biomarker for cancer diagnosis. The serum expression of FAM83H-AS1 was measured by RT-qPCR, which showed a relatively high expression of FAM83H-AS1 in patients with PDAC compared with patients with benign nonpancreatic disease (Fig. 1D). For the serum expression of FAM83H-AS1, there were no significant differences between PDAC and benign pancreatic diseases, similar to the results between patients with benign nonpancreatic disease and patients with benign pancreatic disease (Figure S7). We also detected FAM83H-AS1 expression in PDAC cell lines by RT-qPCR and observed significantly higher FAM83H-AS1 expression in PDAC cell lines (MIA PaCa-2, PANC-1, SW 1990, AsPC-1, BxPC-3 and Capan-2) than in HPDE cells (Fig. 1E). Additionally, the Kaplan–Meier survival analysis revealed that a higher level of FAM83H-AS1 expression was related to reduced OS in both the TCGA cohort and the Tongji cohort (Fig. 1F,G). One well-known characteristic of pancreatic cancer is the low neoplastic cellularity, which may affect the judgment of the real effects of genes on tumor cells. To improve the precision of the results, we excluded samples with a purity below 60% in the TCGA cohort. The survival results still hinted that high expression of FAM83H-AS1 was correlated with poor OS of patients with PDAC (Figure S8). The clinicopathological characteristics of these patients were summarized in Supplementary file 5: Table S5 and Supplementary file 6: Table S6.

The different distribution of lncRNAs in the cytoplasm and/or in the nucleus generally determine the different mainstream regulatory mechanisms [30]. We conducted nuclear/cytoplasmic fractionation followed by RT-qPCR assays (Fig. 1H) and FISH assays (Fig. 1I) to investigate the subcellular distribution of FAM83H-AS1. The results showed that FAM83H-AS1 was mainly localized in the cytoplasm in both PANC-1 and SW 1990 cells.

### FAM83H-AS1 aggravates PDAC cell proliferation, migration and invasion in vitro and in vivo

**Fig. 2:**
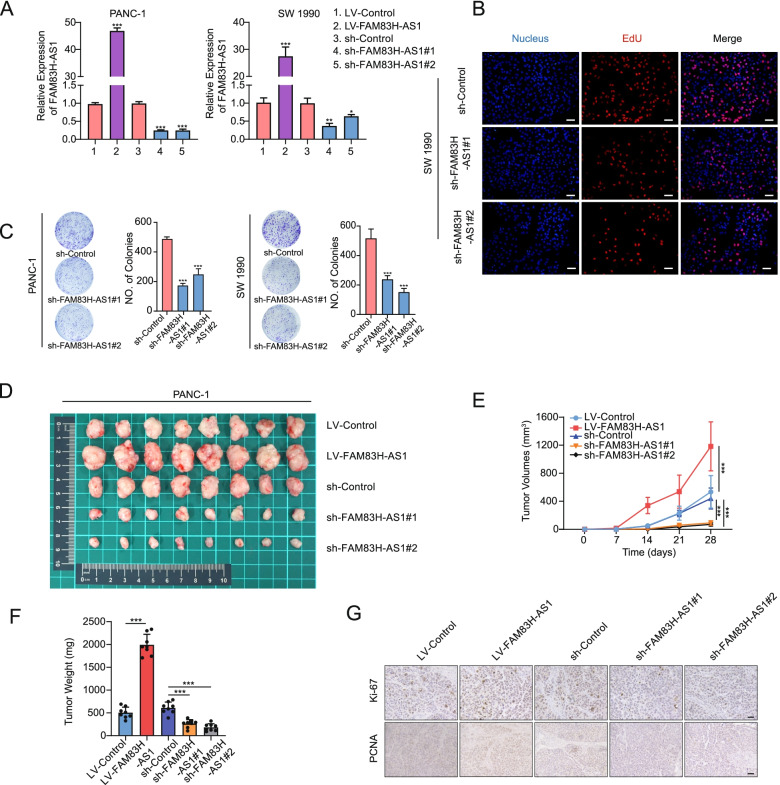
FAM83H-AS1 promotes PDAC cell proliferation in vitro and tumor growth in vivo. **A** The overexpression and knockdown of FAM83H-AS1 in stably transfected PANC-1 and SW 1990 cells was confirmed by RT-qPCR analysis. **B** A combination of EdU and IF assays were used to compare the proliferative activity between the sh-FAM83H-AS1#1/#2 groups and the sh-Control group in SW 1990 cells; scale bar: 50 μm. **C** Colony formation assays showing the colony numbers of tumor cells and analysis of the differences between groups. **D**-**G** A xenograft tumor model was used to analyze the effects of FAM83H-AS1 interference on tumor growth by monitoring the tumor volumes and weight and analyzing the expression of Ki-67 and PCNA in tumor tissue by IHC; scale bar: 100 μm. *P < 0.05, **P < 0.01 and ***P < 0.001

Next, we constructed lentivirus vectors (overexpression and knockdown) and transfected PDAC cell lines. The transfection efficiency in the stable cell lines was verified by RT-qPCR (Fig. 2A). Overexpression of FAM83H-AS1 promoted the proliferation of PANC-1 and SW 1990 cells in vitro, as verified by CCK-8 assays (Figure S9). However, silencing FAM83H-AS1 expression impeded the growth of PDAC cells, as shown by EdU incorporation assays (Fig. 2B) and colony formation assays (Fig. 2C). Also, FAM83H-AS1 overexpression (LV-FAM83H-AS1 group) potently promoted the growth of xenograft tumors compared with that of the negative control group (LV-Control). After the knockdown of FAM83H-AS1 expression (with sh-FAM83H-AS1#1 and sh-FAM83H-AS1#2), the growth of xenograft tumors was reduced (Fig. 2D-F). The tumors were stained by the proliferation-related nuclear antigen Ki-67 and the sensitive marker of proliferative activity PCNA, which demonstrated that FAM83H-AS1 overexpression promoted tumor growth, while the loss of FAM83H-AS1 exerted the opposite effect (Fig. 2G). Overall, these data indicated that FAM83H-AS1 played a crucial role in promoting the proliferation of PDAC cells.

**Fig. 3:**
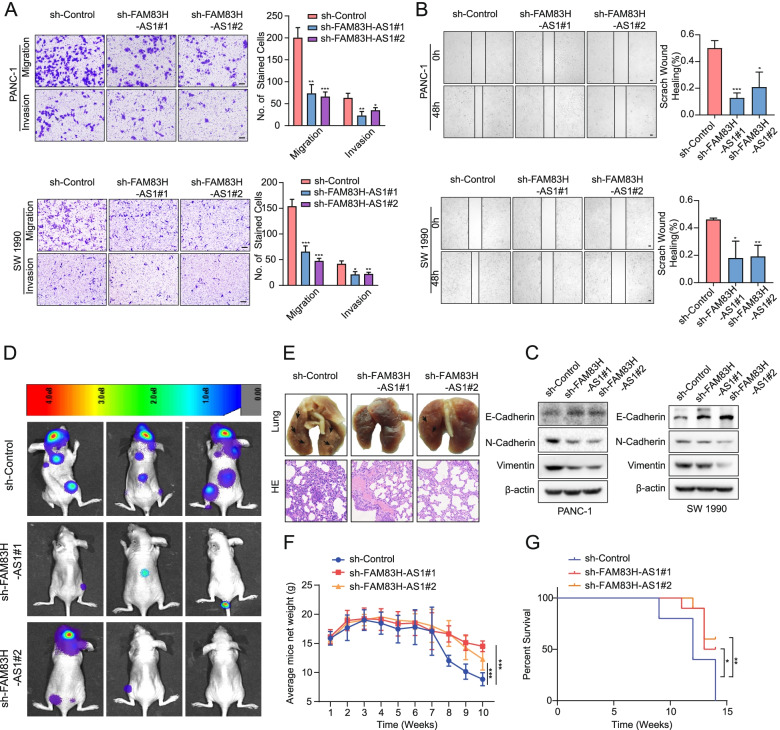
FAM83H-AS1 promotes PDAC cell invasion and metastasis in vitro and in vivo. **A** Transwell assays were conducted to determine the invasion and migration of FAM83H-AS1 shRNA-transfected PDAC cells; scale bar: 100 μm. **B** Wound healing assays were conducted to evaluate the migration of FAM83H-AS1 shRNA-transfected PDAC cells; scale bar: 100 μm. **C** Western blotting was performed to assess the expression of E-cadherin, N-cadherin and Vimentin in PDAC cells after FAM83H-AS1 knockdown. **D**, **E** The mouse model was established by tail vein injection of SW 1990 cells with FAM83H-AS1 knockdown. Representative bioluminescence images (D), representative images of the lungs (E, upper panel) and H&E staining of lung tissues (E, lower panel) are shown. **F** The average weight curve was constructed to assess the effect of the metastasis of PDAC cells with FAM83H-AS1 knockdown on the nutritional condition and growth of mice. **G** Survival analysis was performed on mice bearing the indicated PDAC cells immediately after injection. *P < 0.05, **P < 0.01 and ***P < 0.001

Since FAM83H-AS1 was shown to be related to PDAC metastasis by the screening models, we further examined the effect of FAM83H-AS1 on the migration and invasion of PDAC cells. Transwell and scratch wound-healing assays showed that FAM83H-AS1 knockdown mitigated the migration and invasion of PANC-1 and SW 1990 cells (Fig. 3A, B). An analysis of EMT marker expression by Western blotting showed that the level of the epithelial marker E-cadherin was increased [31–33], but the levels of the mesenchymal markers N-cadherin and Vimentin were decreased in the PDAC cells with FAM83H-AS1 knockdown (Fig. 3C). To establish a systemic metastasis model, we injected SW 1990 cells into nude mice via the tail vein (Fig. 3D). After knockdown of FAM83H-AS1, the number of metastases was significantly decreased compared with that in the sh-Control group (Fig. 3E). The rates of weight loss in the sh-FAM83H-AS1 groups were significantly lower than those in the sh-Control group (Fig. 3F). Moreover, the mice in the sh-FAM83H-AS1 groups experienced longer OS times than those in the sh-Control group (Fig. 3G). Collectively, these data indicated that the loss of FAM83H-AS1 hindered the migration and invasion of PDAC cells.

### FAM83H-AS1 protects FAM83H mRNA from degradation to upregulate FAM83H expression

**Fig. 4:**
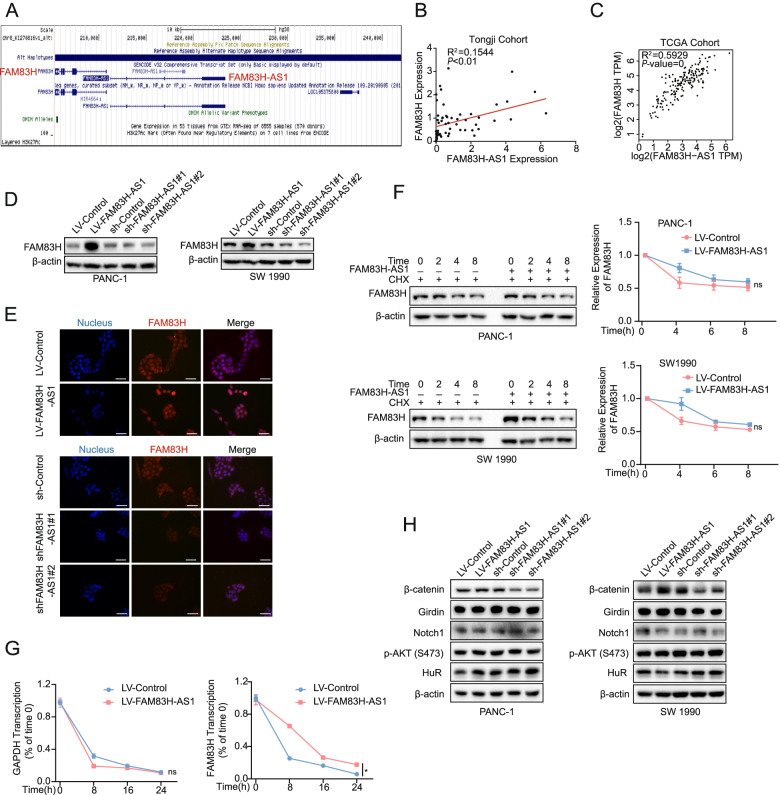
FAM83H-AS1 regulates β-catenin expression and stabilizes FAM83H mRNA in PDAC cells. **A** The unidimensional location diagram of FAM83H-AS1 and the nearby gene FAM83H in the UCSC database. **B**, **C** The positive correlation between FAM83H-AS1 and FAM83H expression in PDAC tissues from the Tongji Hospital cohort (**B**) and TCGA cohort (**C**) was confirmed by Spearman’s correlation analysis. **D**, **E** Western blotting and IF staining were used to assess the expression of FAM83H in PDAC cells upon FAM83H-AS1 overexpression and knockdown; scale bar: 50 μm. **F** FAM83H-AS1 did not affect FAM83H protein stability, as shown by determining the effect of FAM83H-AS1 overexpression on the FAM83H degradation rate after addition of cycloheximide (100 μg/mL). **G** RT-qPCR results showed that FAM83H-AS1 decreased FAM83H mRNA degradation (right panel), while it did not affect GAPDH degradation (left panel) after addition of the RNA synthesis inhibitor actinomycin D (10 μg/mL). **H** Western blot results showed changes in the expression of the key proteins involved in reported mainstream signaling pathways upon FAM83H-AS1 overexpression and knockdown. *P < 0.05

As reported previously, many antisense lncRNAs exert their regulatory effects by interacting with neighboring genes, especially their homologous sense genes [34–37]. Intriguingly, FAM83H-AS1 is the antisense transcript of FAM83H, which has been identified as an essential protein for amelogenesis and has recently been described as a coding gene involved in the progression of cancers [38]. These two genes share a promoter region but are transcribed in opposite directions [39], as shown in the UCSC Genome Browser database (Fig. 4A). The expression levels of both FAM83H-AS1 and FAM83H in PDAC tissues were higher than those in adjacent tissues and exhibited a positive correlation (Fig. 4B). The positive correlation between FAM83H-AS1 and FAM83H expression was also verified in the TCGA database (Fig. 4C). The FAM83H protein level was correspondingly changed upon interference with FAM83H-AS1 expression in both PANC-1 and SW 1990 cells (Fig. 4D). Moreover, IF assays produced similar results (Fig. 4E).

Next, we inhibited protein synthesis in PDAC cells by treatment with cycloheximide and found that FAM83H-AS1 overexpression failed to alter the degradation rates of FAM83H, indicating that the stability of the FAM83H protein was not affected by FAM83H-AS1 (Fig. 4F). Thus, we hypothesized that FAM83H-AS1 might modulate the stability of FAM83H mRNA to regulate the expression of FAM83H. We verified this hypothesis by conducting mRNA degradation assays with actinomycin D (Act D) to inhibit the synthesis of mRNA and found that FAM83H-AS1 indeed protected FAM83H mRNA from degradation (Fig. 4G). Based on these results, FAM83H-AS1 regulated the expression of FAM83H at the mRNA level, which subsequently affected FAM83H protein expression.

Western blotting was conducted to detect the key molecules that participate in the reported regulatory networks of FAM83H-AS1 in other cancers and to elucidate the signaling pathways in which FAM83H-AS1 is involved during PDAC progression. When FAM83H-AS1 was upregulated and downregulated, β-catenin expression was correspondingly increased and decreased, respectively (Fig. 4H). Furthermore, studies have shown that FAM83H is involved in the regulation of β-catenin and the progression of osteosarcomas and gastric carcinoma [40, 41]. These relevant studies stimulated our interest in elucidating the regulatory relationship among these 3 molecules.

### FAM83H exacerbates the proliferation, migration, and invasion of PDAC cells

**Fig. 5:**
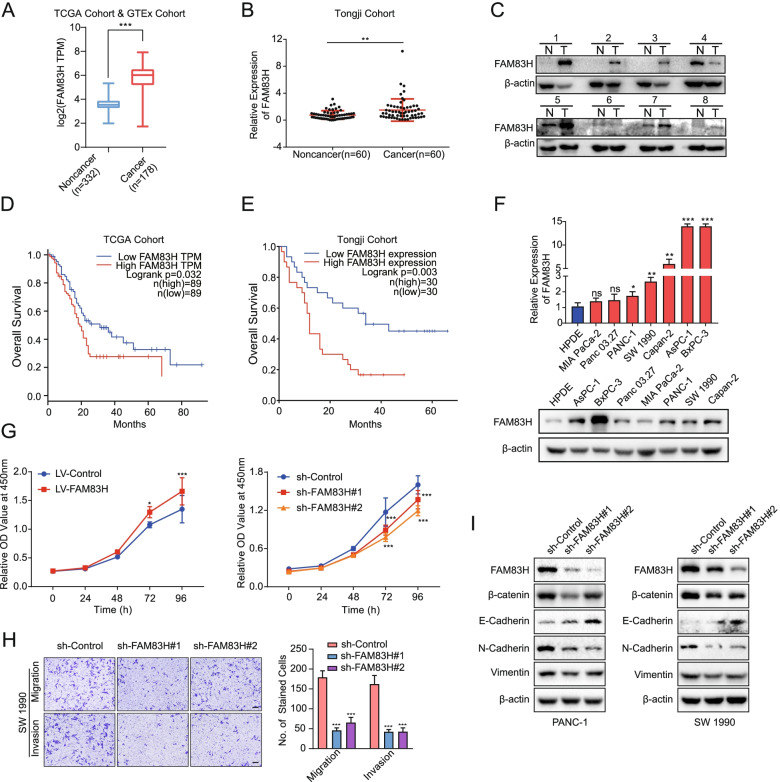
FAM83H is upregulated in PDAC tissues and cells and promotes PDAC progression. **A**, **B** Expression of FAM83H in the combined TCGA and GTEx cohort (**A**) and the cohort from Tongji Hospital (**B**). **C** Western blot results showing that FAM83H was expressed at higher levels in PDAC tissues than in adjacent tissues. **D**, **E** Kaplan–Meier analysis and long-rank test determining the prognostic significance of FAM83H in PDAC patients from the TCGA cohort (**D**) and Tongji Hospital cohort (**E**), respectively. **F** RT–qPCR and Western blot results showing FAM83H expression in HPDE and PDAC cell lines (Panc 03.27, MIA PaCa-2, PANC-1, SW 1990, AsPC-1, BxPC-3 and Capan-2). **G** CCK-8 assays were performed to compare the cell viability between the LV-FAM83H group and the LV-Control group and between the sh-FAM83H#1/#2 group and the sh-Control group. **H** Transwell assays were used to determine the invasion and migration of FAM83H shRNA-transfected PDAC cells. scale bar: 100 μm; **I** Western blots showing the expression of β-catenin, E-cadherin, N-cadherin and Vimentin in PDAC cells with FAM83H knockdown. *P < 0.05, **P < 0.01 and ***P < 0.001

As described above, FAM83H mRNA level was positively correlated with FAM83H-AS1 mRNA level. FAM83H was expressed at higher levels in PDAC tissues than in nontumor tissues from patients in both the combined cohort (Fig. 5A) and the Tongji cohort (Fig. 5B). A higher level of FAM83H was also detected in 8 PDAC tissues compared with the corresponding adjacent tissues by Western blot analysis (Fig. 5C). The Kaplan–Meier analysis showed that patients with PDAC presenting a higher level of FAM83H experienced shorter OS in both TCGA cohort (Fig. 5D) and Tongji cohort (Fig. 5E). Furthermore, RT-qPCR (Fig. 5F, upper panel) and Western blot (Fig. 5F, lower panel) results both showed higher FAM83H expression in PDAC cell lines (AsPC-1, BxPC-3, Panc 03.27, MIA PaCa-2, PANC-1, SW 1990, and Capan-2) than in the HPDE cell line. Collectively, these results indicated that FAM83H expression was positively correlated with PDAC aggressiveness.

We performed a series of functional assays in vitro to explore whether FAM83H altered the biological behavior of PDAC cells. FAM83H was upregulated and downregulated in SW 1990 cells (Figure S10). The CCK-8 assays showed that FAM83H overexpression facilitated the proliferation of SW 1990 cells, while the loss of FAM83H exerted the opposite effect (Fig. 5G). In transwell assays, FAM83H deficiency mitigated the migration and invasion of SW 1990 cells (Fig. 5H). Furthermore, FAM83H knockdown impeded EMT in both PANC-1 and SW 1990 cells by modulating the expression of E-cadherin, N-cadherin and Vimentin (Fig. 5I). Taken together, these findings indicated that FAM83H promoted the progression of PDAC and might play a role similar to that of FAM83H-AS1 in the progression of PDAC.

### FAM83H interacts with β-catenin and protects it from degradation to activate the Wnt/β-catenin signaling pathway

**Fig. 6:**
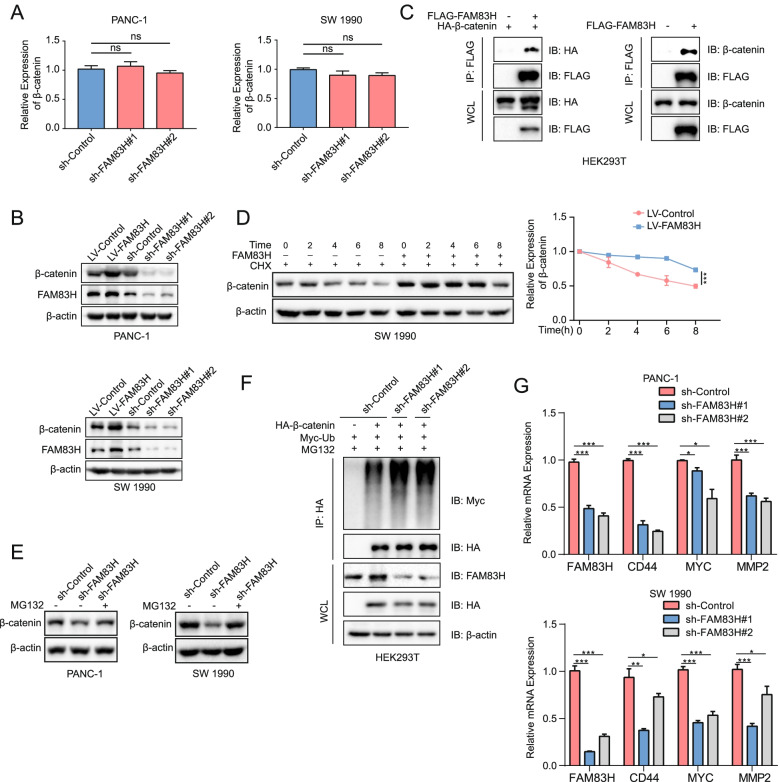
FAM83H binds to β-catenin and protects it from degradation to induce the expression of the effector genes of Wnt/β-catenin signaling. **A** RT-qPCR results showing that the β-catenin mRNA level was not altered by FAM83H knockdown in either PANC-1 or SW 1990 cells. **B** Western blot showing that the β-catenin protein level was synchronously changed upon FAM83H overexpression and knockdown. **C** FAM83H binds to exogenously expressed (left panel) or endogenous β-catenin (right panel). HEK293T cells were transfected with the indicated plasmids, followed by IP with an anti-FLAG antibody and IB with the indicated antibodies. WCL: whole-cell lysate. **D** FAM83H affected β-catenin protein stability, which was assessed by determining the effect of FAM83H overexpression on the β-catenin degradation rate after adding cycloheximide (100 μg/mL). **E** Western blot results showing that the expression of β-catenin in the FAM83H-downregulated PDAC cells treated with the proteasome inhibitor MG132 (10 μM) for 6 h was recovered compared with that in the FAM83H-downregulated PDAC cells. **F** Western blot results showing the change in the ubiquitination of β-catenin. HEK293T cells were cotransfected with HA-β-catenin, sh-FAM83H#1/#2 and MYC-Ub and then treated with 10 μM MG132 for 6 h. Polyubiquitinated proteins were immunoprecipitated with anti-HA beads, followed by immunoblotting with antibodies against MYC and HA. **G** The expression of the effector gene Wnt/β-catenin signaling upon FAM83H knockdown was determined by RT-qPCR; *P < 0.05, **P < 0.01 and ***P < 0.001

**Fig. 7:**
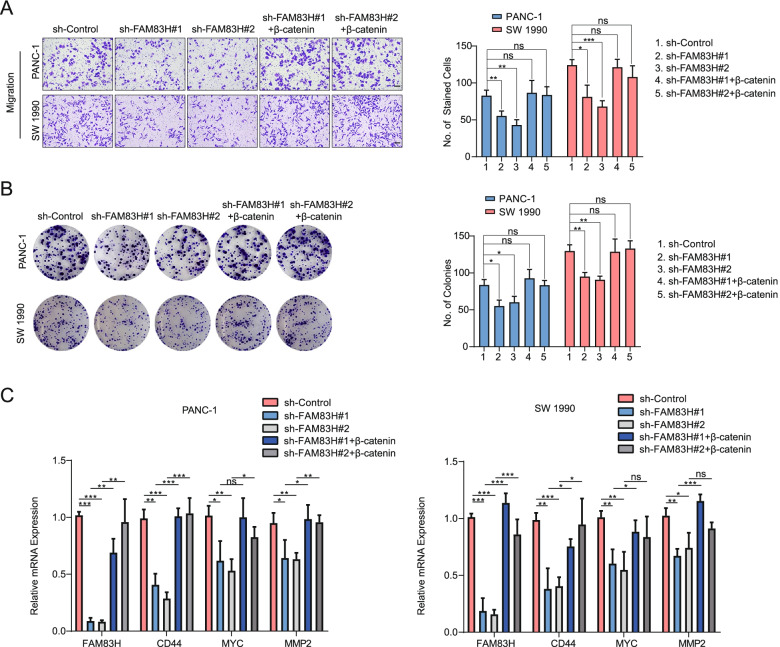
FAM83H regulates PDAC progression via β-catenin. PDAC cells were treated with sh-Control, sh-FAM83H#1, sh-FAM83H#2, sh-FAM83H# 1 + β-catenin or sh-FAM83H#2 + β-catenin. **A** Transwell assays were conducted to assess the migration of cells in each group; scale bar: 100 μm. **B** Colony formation assays were performed to evaluate the proliferation of cells in each group. **C** The expression of effector genes of Wnt/β-catenin signaling in each group was determined by RT-qPCR. *P < 0.05, **P < 0.01 and ***P < 0.001

The β-catenin mRNA level was not affected by the loss of FAM83H (Fig. 6A). Additionally, under the conditions of FAM83H overexpression and knockdown, the expression of β-catenin changed synchronously (Fig. 6B), indicating that FAM83H altered the expression of β-catenin at the post-transcriptional level but not at the transcriptional level. HEK293T cells were transiently transfected with plasmids expressing FLAG-FAM83H and/or HA-β-catenin, and immunoprecipitation was then performed to detect exogenous (Fig. 6C, left panel) and endogenous β-catenin (Fig. 6C, right panel) as a method to determine the interaction between FAM83H and β-catenin. FAM83H interacted with β-catenin. In cells incubated with cycloheximide to inhibit new protein synthesis, the rate of β-catenin degradation was mitigated by the overexpression of FAM83H in SW 1990 cells (Fig. 6D). Moreover, in the cells treated with MG132, a proteasome inhibitor, the decrease in β-catenin levels was reversed by FAM83H knockdown (Fig. 6E). These results showed that FAM83H bound to β-catenin to protect it from proteasomal degradation. Furthermore, HEK293T cells were cotransfected with an shRNA targeting FAM83H, HA-β-catenin and MYC-ubiquitin and were subsequently treated with MG132 for 6 h to determine whether FAM83H impaired β-catenin ubiquitylation. The ubiquitylation status of β-catenin was examined by Western blotting. The results showed that the loss of FAM83H increased β-catenin polyubiquitylation (Fig. 6F). We estimated the expression levels of downstream target genes of β-catenin by performing RT-qPCR to further investigate the effect of FAM83H on the activation of Wnt/β-catenin signaling (Fig. 6G). MYC, CD44 and MMP2 were downregulated in both PANC-1 and SW 1990 cells with FAM83H knockdown. A series of rescue assays was conducted to verify that the regulatory function of FAM83H in PDAC was mediated by β-catenin. In transwell and colony formation assays, FAM83H knockdown (sh-FAM83H#1/sh-FAM83H#2) and simultaneous β-catenin overexpression reversed the decreases in the migration and proliferation of cells in the sh-FAM83H groups (Fig. 7A, B). The rescue assays also showed that the decreased levels of downstream target genes of β-catenin were reversed by knocking down FAM83H (sh-FAM83H#1/sh-FAM83H#2) while simultaneously overexpressing β-catenin, which was verified by RT-qPCR (Fig. 7C). Furthermore, we conducted nuclear/cytosol fractionation Western blot assays to illustrate the translocation of β-catenin affected by FAM83H-AS1 and FAM83H. The results suggested that in both the cytoplasm and he nucleus, β-catenin was upregulated following FAM83H-AS1 overexpression, and this effect was reversed by knocking down FAM83H (Figure S11). Taken together, our data suggested that FAM83H bound to β-catenin to protect it from ubiquitylation-mediated degradation, which subsequently promoted the activation of Wnt/β-catenin signaling pathway.

### FAM83H-AS1 modulates the progression of PDAC through the FAM83H/β-catenin axis

**Fig. 8:**
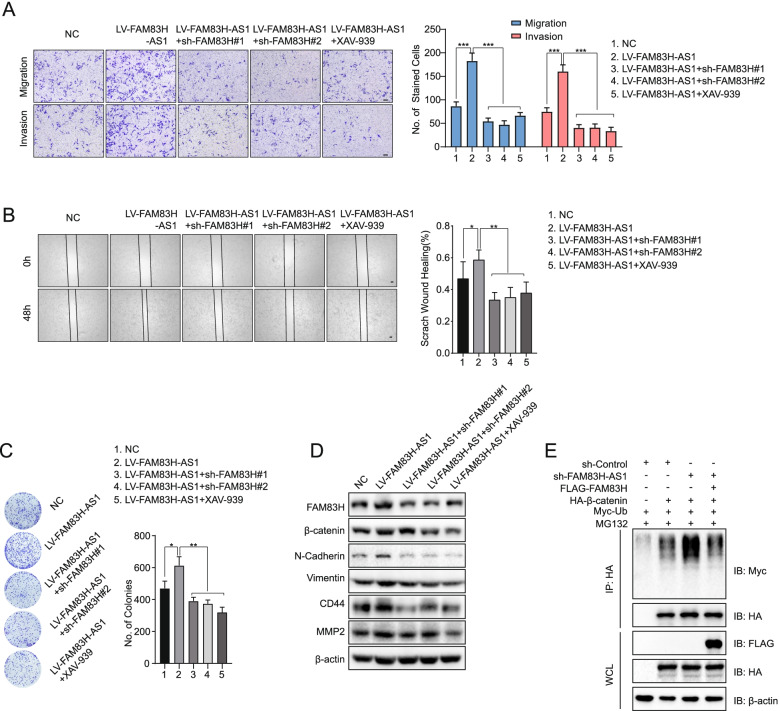
FAM83H-AS1 regulates PDAC progression via FAM83H/Wnt/β-catenin signaling. PDAC cells were treated with NC, LV-FAM83H-AS1, LV-FAM83H-AS1 + sh-FAM83H#1, LV-FAM83H-AS1 + sh-FAM83H#2 and LV-FAM83H-AS1 + VAX-939. **A**, **B** Transwell and wound-healing assays were conducted to assess the invasion and migration of the indicated groups of SW 1990 cells; scale bar: 100 μm. **C** Colony formation assays were used to evaluate the proliferation of the indicated groups of SW 1990 cells. **D** Western blotting was conducted to determine the expression of FAM83H, β-catenin, N-cadherin, Vimentin, CD44 and MMP2. **E** HEK293T cells were cotransfected with HA-β-catenin, FLAG-FAM83H, sh-FAM83H-AS1 and MYC-Ub and then treated with 10 μM MG132 for 6 h. Polyubiquitinated proteins were immunoprecipitated with anti-HA beads, followed by immunoblotting with antibodies against MYC and HA. *P < 0.05, **P < 0.01 and ***P < 0.001

We investigated whether FAM83H-AS1/FAM83H/β-catenin signaling was involved in the progression of PDAC by conducting rescue assays. First, the results of the functional assays showed that the effects of FAM83H-AS1 on promoting cell proliferation, migration and invasion were counteracted by FAM83H knockdown or treatment with an inhibitor of Wnt/β-catenin signaling, namely, XAV-939 (Fig. 8A-C). Next, the expression levels of FAM83H, β-catenin, N-cadherin, Vimentin, CD44 and MMP2 were increased upon FAM83H-AS1 overexpression, and these increases were reversed by either FAM83H knockdown or XAV-939 treatment (Fig. 8D). Furthermore, HEK293T cells were cotransfected with an shRNA targeting FAM83H-AS1, HA-β-catenin and MYC-ubiquitin and were subsequently transfected with FLAG-FAM83H. The cells were then treated with MG132 for 6 h, and the ubiquitylation status of β-catenin was examined by Western blotting. Loss of FAM83H-AS1 promoted β-catenin polyubiquitylation, but this increase in β-catenin polyubiquitylation was reversed by the upregulation of FAM83H (Fig. 8E). In conclusion, FAM83H-AS1 modulated the progression of PDAC through the FAM83H/β-catenin axis.

## Discussion

The early invasion and metastasis of PDAC make timely and effective treatment difficult. A better understanding of the molecular regulatory networks involved in the carcinogenesis, invasion, metastasis, angiogenesis, stem cell-like properties and cancer microenvironment of PDAC would provide us with new prospects and new treatment strategies to constantly improve the survival and quality of life of patients with PDAC [42–44]. In the present study, FAM83H-AS1 expression was positively correlated with shorter survival of PDAC patients and promoted the proliferation, migration, and invasion of PDAC cells through the FAM83H-AS1/FAM83H/β-catenin axis.

LncRNAs, which present a large number of transcripts, are involved in many processes regulating gene expression, including gene transcription, alternative splicing, translation and gene modification [45]. Dysfunction of lncRNAs in these processes leads to various diseases, such as Alzheimer’s disease, neurological diseases, fragile X syndrome and cancer [46]. Accumulating evidence suggests an indispensable role for lncRNAs in the progression of human cancers due to their low sequence conservation, high tissue specificity and functional diversity [47]. Based on the related clinical issues, we screened for metastasis-related lncRNAs and revealed their underlying mechanisms in the regulation of PDAC progression. The three screening models using data from the TCGA database focused on 6 lncRNAs that were related to metastasis in PDAC. Through functional assays and survival analysis, we found that FAM83H-AS1 was the most potent lncRNA promoting the metastasis of PDAC cells.

Luis et al. obtained RNA-seq data after targeting FAM83H-AS1 with two siRNAs in AsPC-1 cells [39]. The gene set enrichment analysis (GSEA) results for the gene signature based on these data indicated that FAM83H-AS1 might be correlated with PDAC metastasis [39]. First, we observed high FAM83H-AS1 expression in PDAC tissues and cell lines, and the subcellular localization of FAM83H-AS1 in the cytoplasm suggested that it might be involved in post-transcriptional regulation. In recent years, with in-depth investigation of ncRNAs, especially lncRNAs, we have identified their vital roles in the pathological process of cancers. In addition to the aberrant expression of lncRNAs detected in solid tumors, many lncRNAs have also been detected in biological fluids, such as serum. With the increasing requirements for easily applied and noninvasive methods for determining clinical diagnosis, we evaluated the expression of FAM83H-AS1 in serum samples and detected a relatively high level in patients with PDAC compared with patients with benign nonpancreatic disease. The survival analysis showed that FAM83H-AS1 was correlated with shorter OS in PDAC patients. Functional assays showed that FAM83H-AS1 promoted the proliferation, migration and invasion of PDAC cells in vitro. Moreover, the upregulation of E-cadherin and downregulation of N-cadherin/Vimentin observed upon interference with FAM83H-AS1 expression were consistent with the GSEA results, indicating that FAM83H-AS1 plays a role in EMT in PDAC. The xenograft tumor model and systemic metastasis model in nude mice produced similar results to those of our in vitro assays. FAM83H-AS1, which is located on chromosome 8 (8q23.3-8q24.3), is the antisense transcript of FAM83H, and the two genes have a potential intrinsic regulatory relationship because they share the same promoter region and are transcribed in opposite directions [39]. In our study, FAM83H-AS1 expression was positively correlated with FAM83H expression at both the mRNA and protein levels. This finding further suggested that FAM83H-AS1 protected FAM83H mRNA but not the FAM83H protein from degradation in PDAC cells. In terms of the mechanism, we investigated the signaling pathways in which FAM83H-AS1 participates in other cancers. Ma et al. revealed that FAM83H-AS1 promoted cell proliferation, migration and invasion in hepatocellular carcinoma by targeting the Wnt/β-catenin pathway [48]. Feng et al. reported that FAM83H-AS1 facilitated the progression of esophageal squamous cell carcinoma via the miR-10a-5p/Girdin axis [49]. Dou et al. reported that by stabilizing the HuR protein, FAM83H-AS1 contributed to radioresistance, proliferation, and metastasis in ovarian cancer [50]. According to Zhang et al., FAM83H-AS1 promoted the proliferation and invasion of lung cancer via EGFR, AKT and ERK1/2 [51]. In the study by Wei et al., nucleus pulposus cell growth was induced by FAM83H-AS1 targeting the Notch signaling pathway [52]. The key molecules in the signaling pathways mentioned above were detected in PDAC cells with FAM83H-AS1 interference, and only β-catenin exhibited a corresponding change in expression. Interestingly, previous studies showed that FAM83H promoted the progression of osteosarcoma and gastric carcinoma by regulating β-catenin [40, 41]. Therefore, the underlying interactive relations among FAM83H-AS1, FAM83H and β-catenin were of interest to us.

**Fig. 9:**
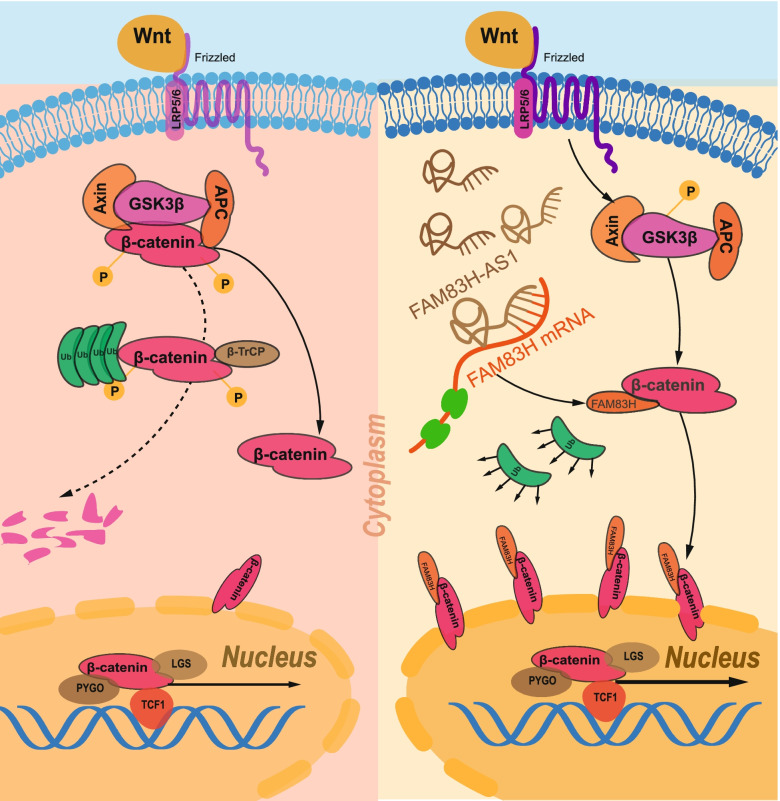
Graphic illustration of the FAM83H-AS1/FAM83H/β-catenin regulatory axis in PDAC. Initially, FAM83H-AS1 induced FAM83H expression by stabilizing its mRNA. Upregulated FAM83H bound to β-catenin to protect it from degradation and then induced the expression of effector genes of Wnt/β-catenin signaling

In PDAC cells, FAM83H exerted the same effects as FAM83H-AS1 on the progression of PDAC, according to the results of the functional assays. Additionally, FAM83H affected the protein level, rather than the mRNA level, of β-catenin by binding β-catenin and delaying its degradation. The interaction between FAM83H and β-catenin was detected by coimmunoprecipitation. After treatment with MG132, the decrease in the β-catenin level caused by FAM83H knockdown was reversed, indicating that FAM83H mainly inhibited the proteasomal degradation of β-catenin. Through coimmunoprecipitation, we found that FAM83H knockdown increased the polyubiquitylation of β-catenin. As a result, FAM83H modulated the activation of Wnt/β-catenin signaling. Furthermore, our rescue assays demonstrated that FAM83H-AS1 promoted the progression of PDAC via the FAM83H/β-catenin axis (Fig. 9).

In summary, FAM83H-AS1 and FAM83H were both upregulated in PDAC and associated with a poor prognosis for patients with PDAC. In PDAC cells, FAM83H-AS1 stabilized FAM83H mRNA to promote the expression of FAM83H, and the accumulated FAM83H then interacted with β-catenin and inhibited its degradation to activate the Wnt/β-catenin signaling pathway. However, further exploration of the precise mechanism by which FAM83H-AS1 regulates the stabilization of FAM83H mRNA is needed. The FAM83H-AS1/FAM83H/β-catenin axis regulates the proliferation, migration and invasion of PDAC cells and might be a novel prognostic indicator and therapeutic target for PDAC.

## Conclusion

In conclusion, our study determined that FAM83H-AS1 and FAM83H were both upregulated in PDAC tissues and were closely correlated with shorter survival of patients with PDAC. Mechanistically FAM83H-AS1 stabilized FAM83H mRNA and enabled FAM83H to protect β-catenin from degradation, by which Wnt/β-catenin signaling was activated. The FAM83H-AS1/FAM83H/β-catenin axis regulates proliferation, invasion and metastasis in PDAC and might be a novel prognostic indicator and therapeutic target for PDAC.

## Supplementary Information

**Additional file 1:** **Table S1.** sh-RNAs / si-RNAs sequences.

**Additional file 2:** **Table S2.** Sequence of primers used in RT-qPCR.

**Additional file 3:** **Table S3.** The basic clinical characteristics of patients in serum test.

**Additional file 4:** **Table S4.** The basic clinical characteristics of patients in tissue test.

**Additional file 5:** **Table S5.** Correlation between FAM83H-AS1 expression and clinicopathological parameters.

**Additional file 6:** **Table S6.** Correlation between FAM83H-AS1 expression and clinicopathological parameters.

**Additional file 7:** **Supplementary Figure 1.** Hierarchical cluster heat map of differentially expressed lncRNAs in PDAC and corresponding normal tissues generated from RNA sequencing data from the TCGA database. Red in the heat map represents upregulation; Green represents downregulation. 817 differentially expressed lncRNAs were screened out in the comparing mode between PDAC and pancreas. **Supplementary Figure 2.** Hierarchical cluster heat map of differentially expressed lncRNAs in T2N0M0 PDAC and T2N1M0/T2N0M1/T2N1M1 PDAC tissues generated from RNA sequencing data from the TCGA database. Red in the heat map represents upregulation; Green represents downregulation. 275 differentially expressed lncRNAs were screened out in the comparing mode between T2N0M0 and T2N1M0/T2N0M1/T2N1M1. **Supplementary Figure 3.** Hierarchical cluster heat map of differentially expressed lncRNAs in T3N0M0 PDAC and T3N1M0/T3N0M1/T3N1M1 PDAC tissues generated from RNA sequencing data from the TCGA database. Red in the heat map represents upregulation; Green represents downregulation. 169 differentially expressed lncRNAs were screened out in the comparing mode between T3N0M0 with T3N1M0/T3N0M1/T3N1M1. **Supplementary Figure 4.** Survival analysis was used to illustrate the relationship between the level of 6 lncRNAs (FAM83H-AS1, LINC00365, LINC00628, LINC00261, AFAP1-AS1, and HNF1A-AS1) and overall survival in TCGA cohort, respectively. **Supplementary Figure 5.** Migration capacities of 6 lncRNAs were estimated in vitro. (A) Transwell assays were used to determine the migration capabilities of 6 lncRNAs siRNA-transfected PDAC cells, respectively; Scale bar: 100 μm. (B) Wound healing assays were conducted to evaluate the migration abilities of 6 lncRNAs siRNA-transfected PDAC cells, respectively; Scale bar: 100 μm. *P < 0.05, **P < 0.01, ***P < 0.001. **Supplementary Figure 6.** Effect of knockdown of 6 candidate lncRNAs on the filamentous state of F-actin. SW 1990 cells were treated with lncRNAs siRNA or control siRNA respectively and analyzed by immunofluorescence, which were estimated the bundling and disassembling sates of F-actin. **Supplementary Figure 7.** Expression of FAM83H-AS1 in serums were tested in the patients with PDAC, benign non-pancreatic disease and benign pancreatic disease from Tongji hospital. **Supplementary Figure 8.** Survival analysis was used to illustrate the relationship between the level of FAM83H-AS1 and overall survival in high purity TCGA cohort, in which the samples below 60% neoplastic cellularity were excluded. **Supplementary Figure 9.** CCK-8 assays were used to compare the cell viability between LV-FAM83H-AS1 group and LV-Control group in PANC-1 and SW 1990 cells. ***P < 0.001. **Supplementary Figure 10.** FAM83H expression in stable FAM83H overexpressed and down-expressed PDAC cells, confirmed by RT-qPCR analysis. *P < 0.05, **P < 0.01, ***P < 0.001. **Supplementary Figure 11.** FAM83H-AS1 promotes β-catenin nuclear location via FAM83H. Western blot for FAM83H, β-catenin, GAPDH, and Histone-H3 with the protein lysates fractionated according to subcellular localization after overexpressing FAM83H-AS1 and knocking down FAM83H in PANC-1 and SW 1990 cells.

## Data Availability

Datasets used and/or analyzed during the current study are available from the corresponding author upon reasonable request.

## Abbreviations

FAM83H-AS1: FAM83H antisense RNA 1
PCNA: Proliferating cell nuclear antigen
lncRNA: Long noncoding RNA
PDAC: Pancreatic ductal adenocarcinoma
TCGA: The Cancer Genome Atlas
F-actin: Filamentous actin
EMT: Epithelial-mesenchymal transition
OS: Overall survival
NC: Negative control
GTEx: Genotype-Tissue Expression
RT-qPCR: Quantitative real-time PCR
FISH: Fluorescence in situ hybridization
