# Supplementary material for: LncRNA FAM83H-AS1 promotes the malignant progression of pancreatic ductal adenocarcinoma by stabilizing FAM83H mRNA to protect β-catenin from degradation
Source: J Exp Clin Cancer Res. 2022 Sep 29;41:288. doi: 10.1186/s13046-022-02491-2 (PMC9520839; doi:10.1186/s13046-022-02491-2)
Supplement: Supplementary file 1 — Additional file 1: Table S1. sh-RNAs / si-RNAs sequences. [file 13046_2022_2491_MOESM1_ESM.docx]

**Supplementary file 1**

**Table S1: sh-RNAs / si-RNAs sequences.**

| **sh-RNAs** | **Sense-sequence** |
| --- | --- |
| **sh-FAM83H-AS1#1** | **5’-CCGGGCTGAATCACGTCAAGTATTCCTCGAGGAATACTTGACGTGATTCAGCTTTTTG-3’** |
| **sh-FAM83H-AS1#2** | **5’-CCGGGCATTCAGCTGGCAGCTAAGACTCGAGTCTTAGCTGCCAGCTGAATGCTTTTTG-3’** |
| **sh-FAM83H#1** | **5’-CCGGGAAGTCCAACTACAGGATTTACTCGAGTAAATCCTGTAGTTGGACTTCTTTTTG-3’** |
| **sh-FAM83H#2** | **5’-CCGGGAGGAAGTCCAACTACAGGATCTCGAGATCCTGTAGTTGGACTTCCTCTTTTTG-3’** |
| **si-RNA** | **Target sequence** |
| **si-LINC00261** | \| **GCAATTAATTCAGGACACT** \| \| --- \| |
| **si-LINC00628** | **GAGGCAAAGTGCATAGCTT** |
| **si-LINC00365** | **GCAGCAGAGCAGTATAAAT** |
| **si-AFAP1-AS1** | **CTCGTTGTGAAACTTAAAT** |
| **si-FAM83H-AS1** | **GCATTCAGCTGGCAGCTAA** |
| **si-HNF1A-AS1** | **CACCTGCATTCAAACTCGGACTGTT** |
