# Supplementary material for: LncRNA FAM83H-AS1 promotes the malignant progression of pancreatic ductal adenocarcinoma by stabilizing FAM83H mRNA to protect β-catenin from degradation
Source: J Exp Clin Cancer Res. 2022 Sep 29;41:288. doi: 10.1186/s13046-022-02491-2 (PMC9520839; doi:10.1186/s13046-022-02491-2)
Supplement: Supplementary file 2 — Additional file 2: Table S2. Sequence of primers used in RT-qPCR. [file 13046_2022_2491_MOESM2_ESM.docx]

**Supplementary file 2**

**Table S2: sequence of primers used in RT-qPCR**

| **Gene** | **Primer sequence** |
| --- | --- |
| ***FAM83H-AS1*** | **F: AAGCAGTGCGTTGAGTGTTC**  **R: CCAGTTCCGACGATGGTGAT** |
| ***FAM83H*** | **F:** **TGCCGCCTCACTACAAAGAG**  **R:** **TCTCGGGTAACATACTGCGGA** |
| ***MMP2*** | **F: GAGGTCGCTTTCTTTGCCATCT**  **R: AGCGACTCCATCTTGAACAGG** |
| ***MYC*** | **F: TTGCTGGGTTATTTTAATCAT**  **R: ACTGTTTGACAAACCGCATCC** |
| ***CD44*** | **F: CTGCCGCTTTGCAGGTGTA**  **R: CATTGTGGGCAAGGTGCTATT** |
| ***GAPDH*** | **F:** **AACAGCGACACCCACTCCTC**  **R:** **GGAGGGGAGATTCAGTGTGGT** |
| ***ACTB*** | **F: CATGTACGTTGCTATCCAGGC**  **R: CTCCTTAATGTCACGCACGAT** |
