# Supplementary material for: LncRNA FAM83H-AS1 promotes the malignant progression of pancreatic ductal adenocarcinoma by stabilizing FAM83H mRNA to protect β-catenin from degradation
Source: J Exp Clin Cancer Res. 2022 Sep 29;41:288. doi: 10.1186/s13046-022-02491-2 (PMC9520839; doi:10.1186/s13046-022-02491-2)
Supplement: Supplementary file 5 — Additional file 5: Table S5. Correlation between FAM83H-AS1 expression and clinicopathological parameters. [file 13046_2022_2491_MOESM5_ESM.docx]

**Supplementary file 5**

**Table S5:**

**Correlation between FAM83H-AS1 expression and clinicopathological parameters**

| n (%) |  | FAM83H-AS1 |  |  |
| --- | --- | --- | --- | --- |
|  |  | Low | High | *P* |
| **Age (years)** |  |  |  |  |
| <50 | 29 (32.6%) | 12 | 17 | 0.290 |
| ≥50 | 70 (78.7%) | 32 | 28 |  |
| **Sex** |  |  |  | 0.421 |
| Male | 57 (64.0%) | 30 | 27 |  |
| Female | 32 (36.0%) | 14 | 18 |  |
| **TNM stage** |  |  |  | 0.297 |
| I | 49 (55.1%) | 23 | 26 |  |
| II | 27 (30.3%) | 12 | 15 |  |
| III | 13 (14.6%) | 9 | 4 |  |
| **Tumor size (cm)** |  |  |  | 0.237 |
| <3 | 49 (55.1%) | 27 | 22 |  |
| ≥3 | 40 (44.9%) | 17 | 23 |  |
| **Lymph node metastasis** |  |  |  | 0.303 |
| N0 | 52 (58.4%) | 24 | 28 |  |
| N1 | 24 (27.0%) | 11 | 13 |  |
| N2 | 13 (14.6%) | 9 | 4 |  |
