# Supplementary material for: LncRNA FAM83H-AS1 promotes the malignant progression of pancreatic ductal adenocarcinoma by stabilizing FAM83H mRNA to protect β-catenin from degradation
Source: J Exp Clin Cancer Res. 2022 Sep 29;41:288. doi: 10.1186/s13046-022-02491-2 (PMC9520839; doi:10.1186/s13046-022-02491-2)
Supplement: Supplementary file 6 — Additional file 6: Table S6. Correlation between FAM83H-AS1 expression and clinicopathological parameters. [file 13046_2022_2491_MOESM6_ESM.docx]

**Supplementary file 6**

**Table S6:**

**Correlation between FAM83H-AS1 expression and clinicopathological parameters**

| n (%) |  | FAM83H-AS1 |  |  |
| --- | --- | --- | --- | --- |
|  |  | Low | High | *P* |
| **Age (years)** |  |  |  |  |
| <50 | 18 (30.0%) | 7 | 11 | 0.260 |
| ≥50 | 42 (70.0%) | 23 | 19 |  |
| **Sex** |  |  |  | 0.584 |
| Male | 40 (66.7%) | 19 | 21 |  |
| Female | 20 (33.3%) | 11 | 9 |  |
| **TNM stage** |  |  |  | 0.158 |
| I | 27 (45.0%) | 17 | 10 |  |
| II | 20 (33.3%) | 7 | 13 |  |
| III | 13 (21.7%) | 6 | 7 |  |
| **Tumor size (cm)** |  |  |  | 0.432 |
| <3 | 35 (58.3%) | 19 | 16 |  |
| ≥3 | 25 (41.7%) | 11 | 14 |  |
| **Lymph node metastasis** |  |  |  | 0.262 |
| N0 | 28 (46.7%) | 17 | 11 |  |
| N1 | 19 (31.7%) | 7 | 12 |  |
| N2 | 13 (21.7%) | 6 | 7 |  |
