# Supplementary figures and images for: LncRNA FAM83H-AS1 promotes the malignant progression of pancreatic ductal adenocarcinoma by stabilizing FAM83H mRNA to protect β-catenin from degradation
Source: J Exp Clin Cancer Res. 2022 Sep 29;41:288. doi: 10.1186/s13046-022-02491-2 (PMC9520839; doi:10.1186/s13046-022-02491-2)

Fig S1

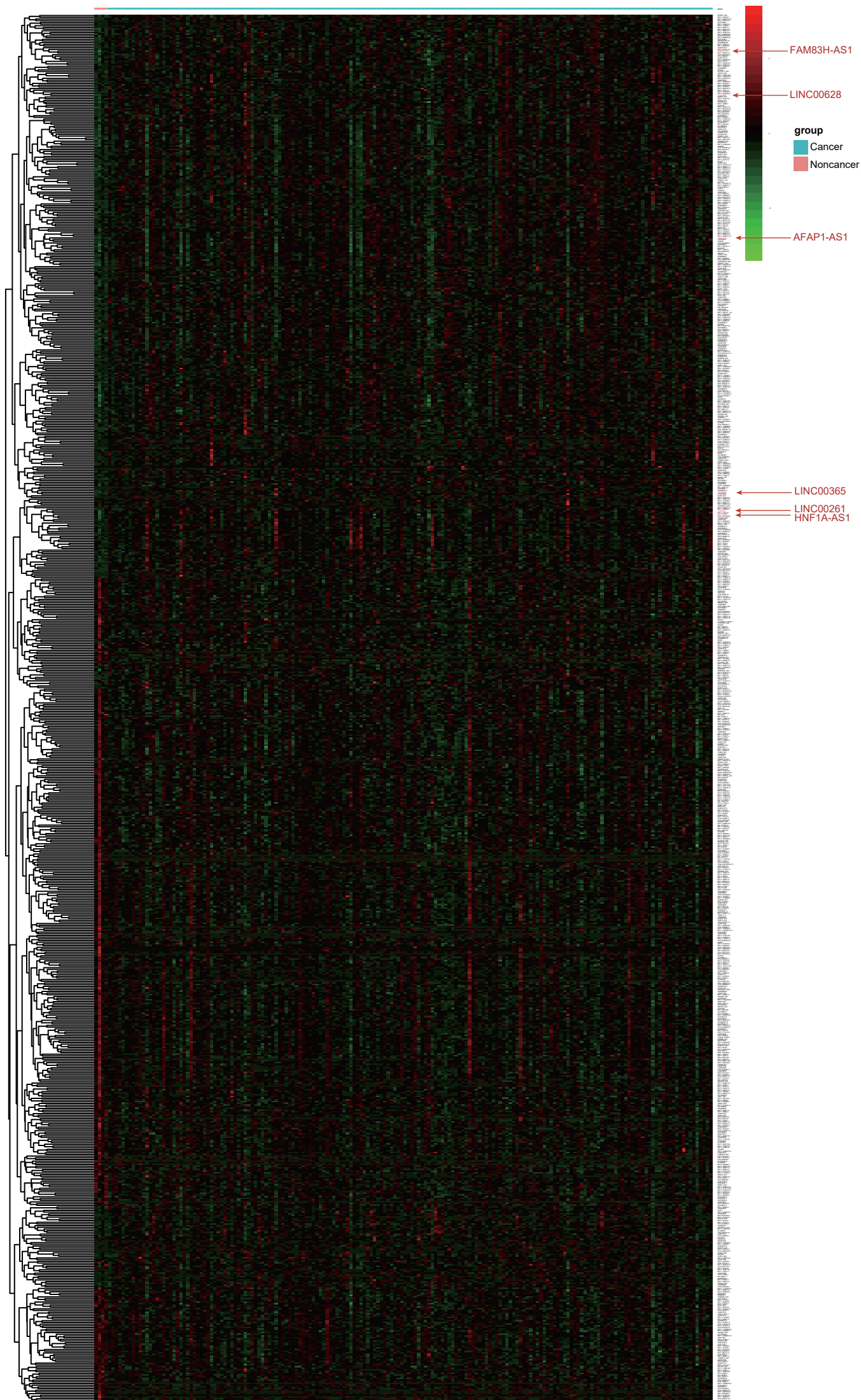

Fig S2

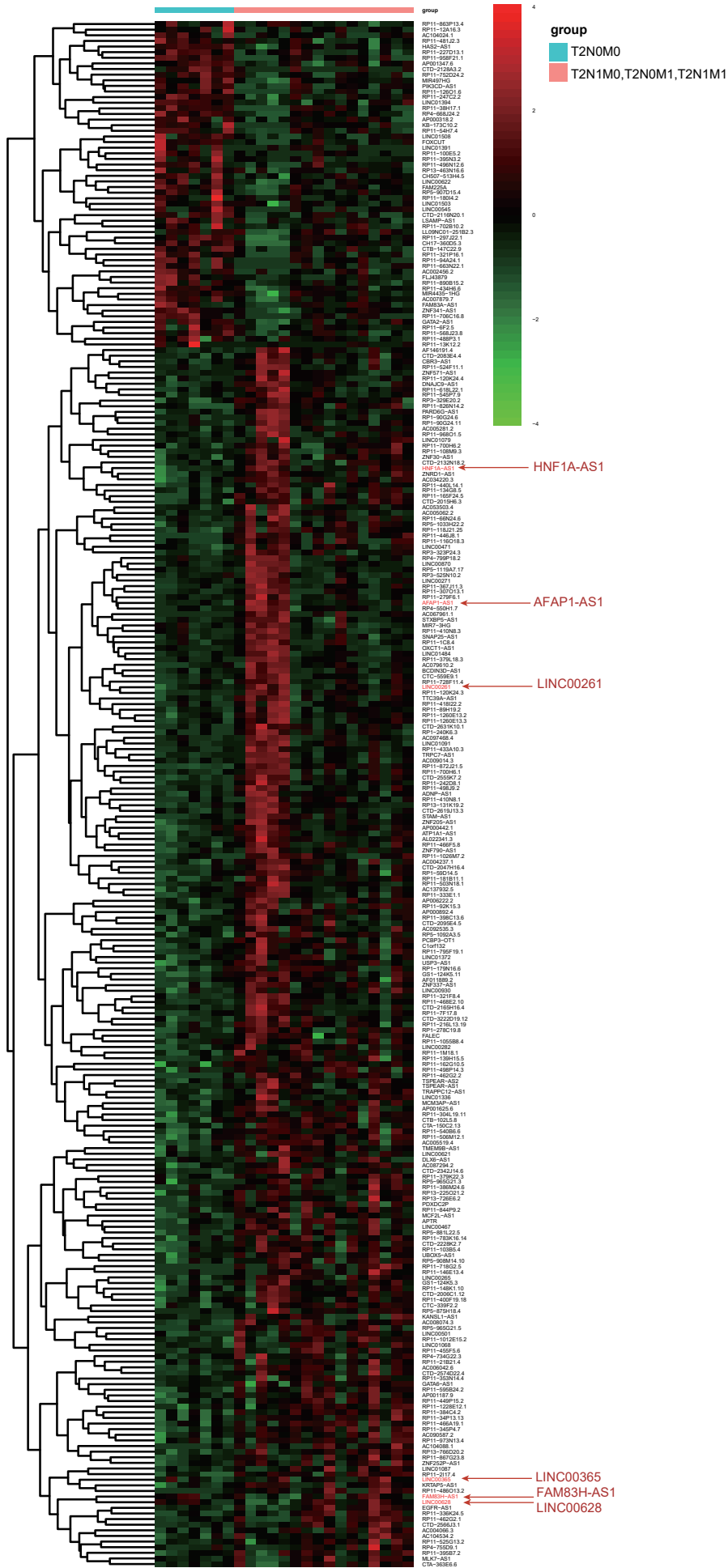

Fig S3

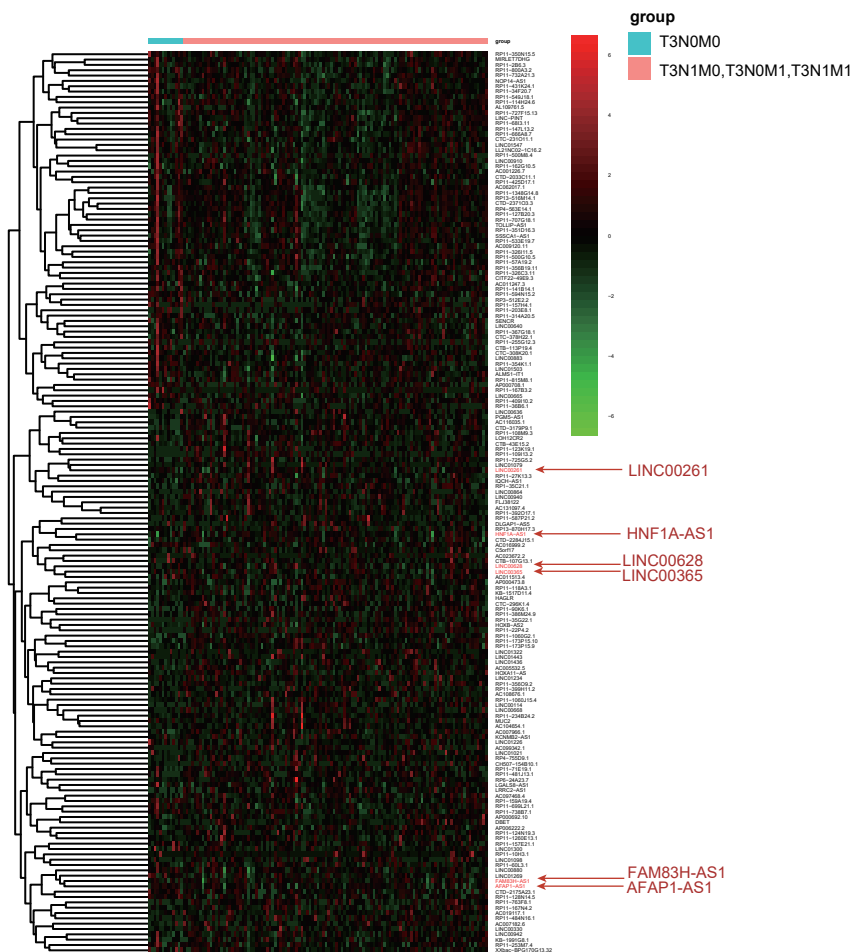

Fig S4

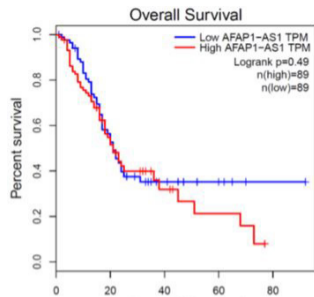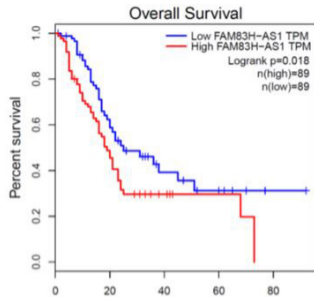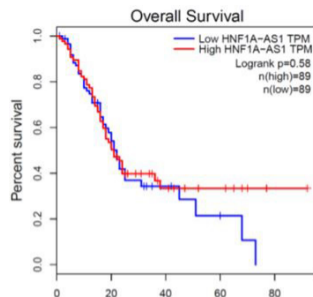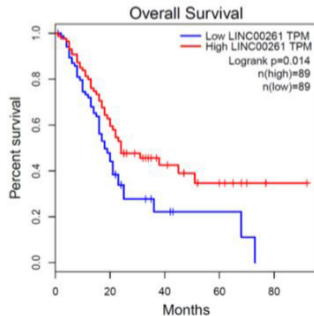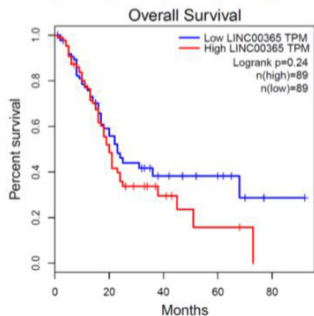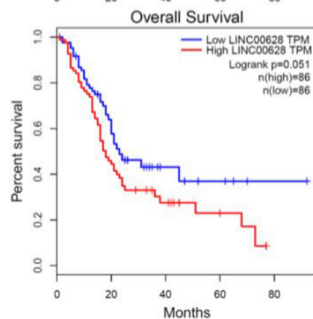

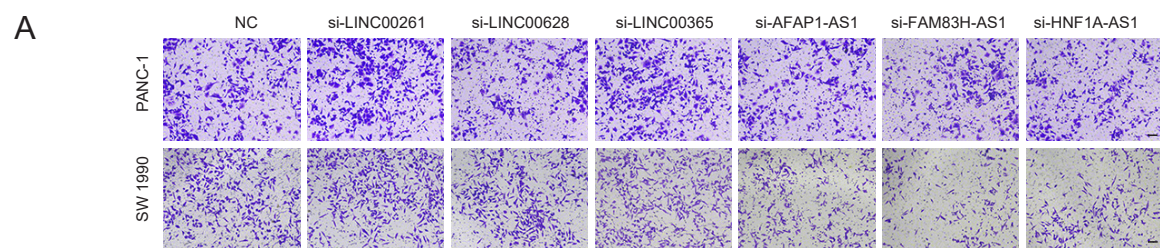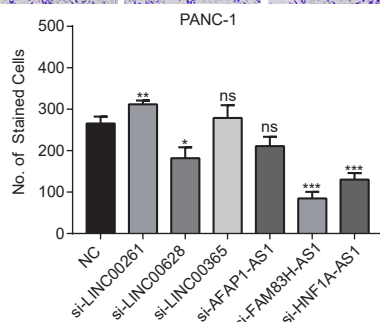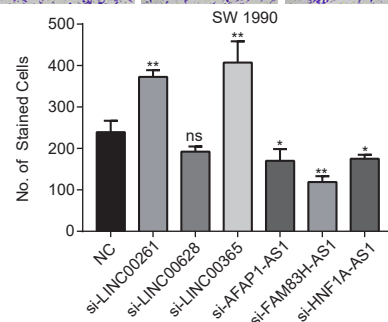

**B**

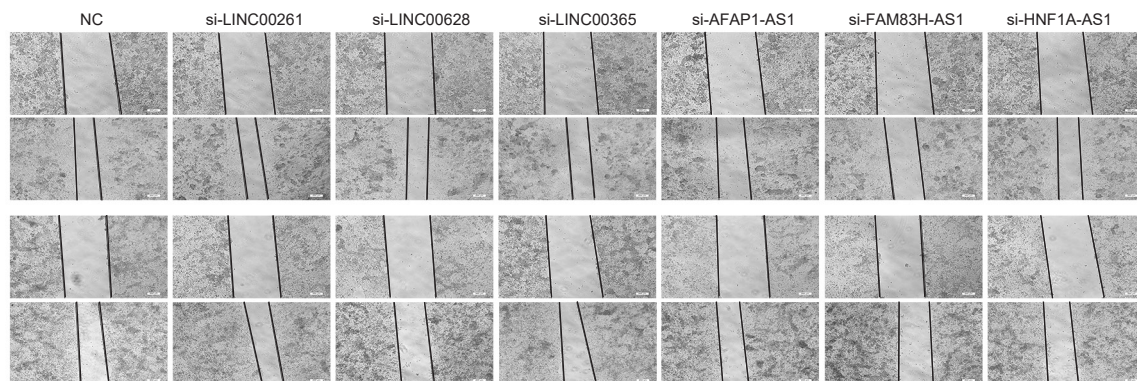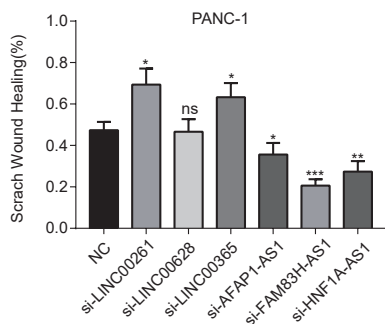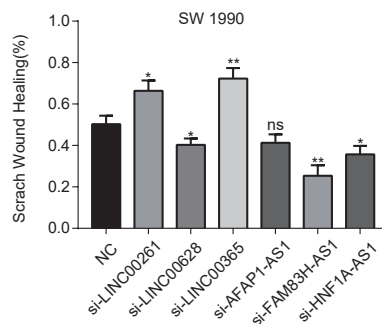

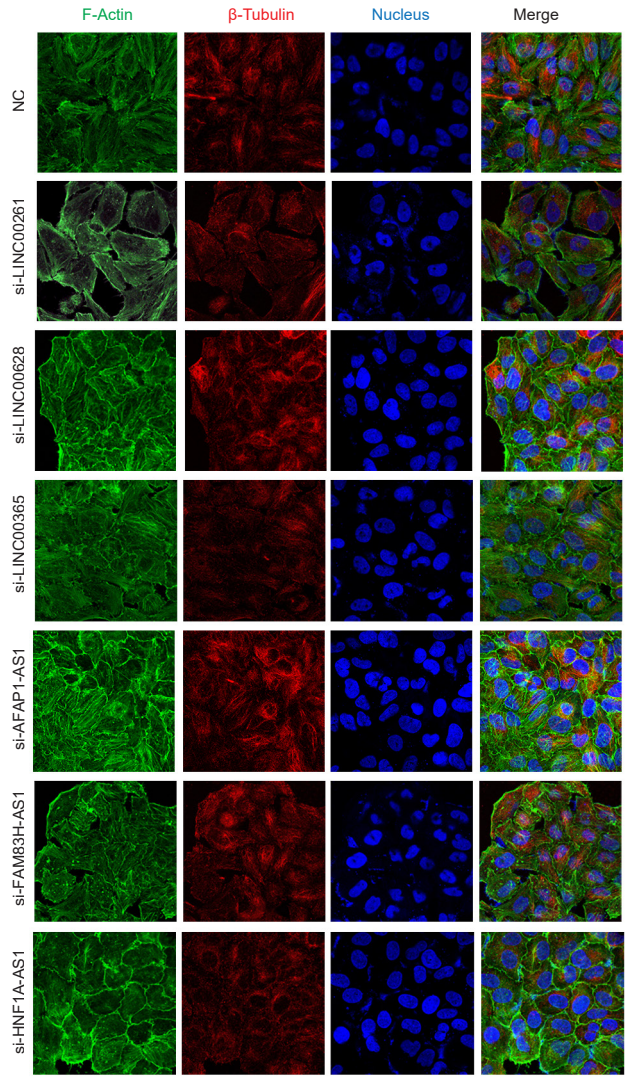

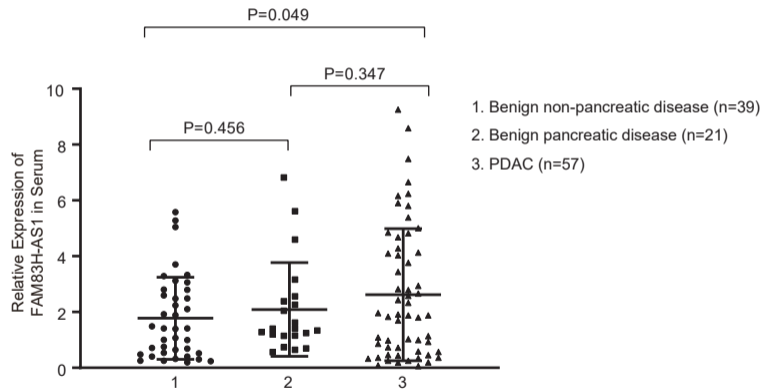

Fig S8

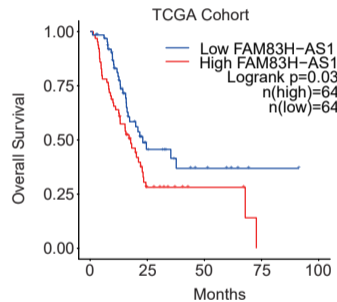

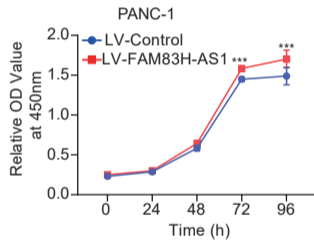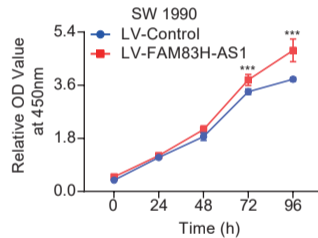

Fig S10

PANC-1

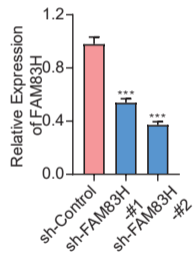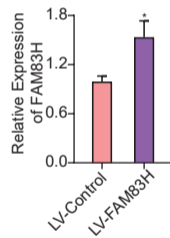

SW 1990

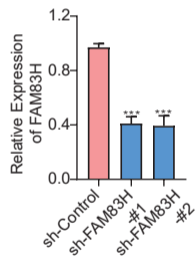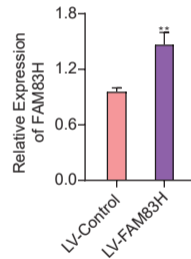

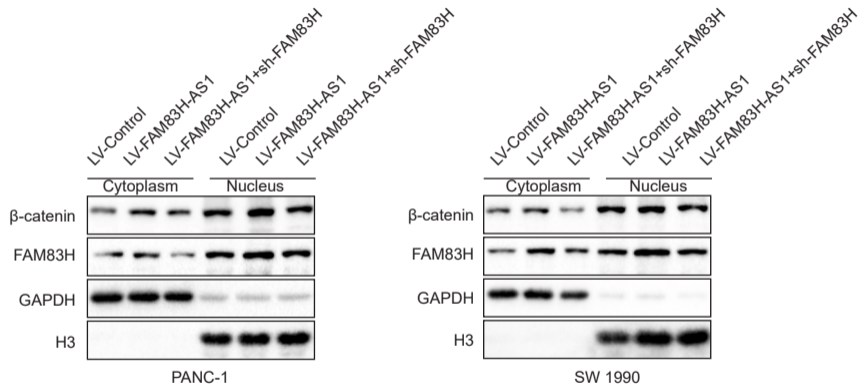

Supplement: Supplementary file 7 — Additional file 7: Supplementary Figure 1. Hierarchical cluster heat map of differentially expressed lncRNAs in PDAC and corresponding normal tissues generated from RNA sequencing data from the TCGA database. Red in the heat map represents upregulation; Green represents downregulation. 817 differentially expressed lncRNAs were screened out in the comparing mode between PDAC and pancreas. Supplementary Figure 2. Hierarchical cluster heat map of differentially expressed lncRNAs in T2N0M0 PDAC and T2N1M0/T2N0M1/T2N1M1 PDAC tissues generated from RNA sequencing data from the TCGA database. Red in the heat map represents upregulation; Green represents downregulation. 275 differentially expressed lncRNAs were screened out in the comparing mode between T2N0M0 and T2N1M0/T2N0M1/T2N1M1. Supplementary Figure 3. Hierarchical cluster heat map of differentially expressed lncRNAs in T3N0M0 PDAC and T3N1M0/T3N0M1/T3N1M1 PDAC tissues generated from RNA sequencing data from the TCGA database. Red in the heat map represents upregulation; Green represents downregulation. 169 differentially expressed lncRNAs were screened out in the comparing mode between T3N0M0 with T3N1M0/T3N0M1/T3N1M1. Supplementary Figure 4. Survival analysis was used to illustrate the relationship between the level of 6 lncRNAs (FAM83H-AS1, LINC00365, LINC00628, LINC00261, AFAP1-AS1, and HNF1A-AS1) and overall survival in TCGA cohort, respectively. Supplementary Figure 5. Migration capacities of 6 lncRNAs were estimated in vitro. (A) Transwell assays were used to determine the migration capabilities of 6 lncRNAs siRNA-transfected PDAC cells, respectively; Scale bar: 100 μm. (B) Wound healing assays were conducted to evaluate the migration abilities of 6 lncRNAs siRNA-transfected PDAC cells, respectively; Scale bar: 100 μm. *P < 0.05, **P < 0.01, ***P < 0.001. Supplementary Figure 6. Effect of knockdown of 6 candidate lncRNAs on the filamentous state of F-actin. SW 1990 cells were treated with lncRNAs siRNA or cont [file 13046_2022_2491_MOESM7_ESM.pdf]
